# Subcutaneous electroencephalography monitoring for people with epilepsy and intellectual disability: co-production workshops

**DOI:** 10.1192/bjo.2024.825

**Published:** 2024-12-13

**Authors:** Edward Meinert, Madison Milne-Ives, Jennifer Sawyer, Liz Boardman, Sarah Mitchell, Brendan Mclean, Mark Richardson, Rohit Shankar

**Affiliations:** Translational and Clinical Research Institute, Newcastle University, Newcastle upon Tyne, UK; and Department of Primary Care and Public Health, School of Public Health, Imperial College London, London, UK; Translational and Clinical Research Institute, Newcastle University, Newcastle upon Tyne, UK; and Centre for Health Technology, School of Nursing and Midwifery, University of Plymouth, Plymouth, UK; Plymouth Dental School, Faculty of Health, University of Plymouth, Plymouth, UK; Cornwall Intellectual Disability Equitable Research (CIDER), Cornwall Partnership NHS Foundation Trust, Bodmin, UK; Cornwall Intellectual Disability Equitable Research (CIDER), Cornwall Partnership NHS Foundation Trust, Bodmin, UK; and Peninsula Medical School, Faculty of Health, University of Plymouth, Plymouth, UK; Peninsula Medical School, Faculty of Health, University of Plymouth, Plymouth, UK; and Department of Neurology, Royal Cornwall Hospitals NHS Trust, Treliske, UK; Department of Basic and Clinical Neuroscience, Institute of Psychiatry Psychology and Neuroscience, King's College London, London, UK

**Keywords:** Intellectual disability, epilepsy, electroencephalography, co-production, patient and public involvement

## Abstract

**Background:**

Nearly 25% of people with intellectual disability (PwID) have epilepsy compared to 1% of the UK general population. PwID are commonly excluded from research, eventually affecting their care. Understanding seizures in PwID is particularly challenging because of reliance on subjective external observation and poor objective validation. Remote electroencephalography (EEG) monitoring could capture objective data, but particular challenges and implementation strategies for this population need to be understood.

**Aim:**

This co-production aimed to explore the accessibility and potential impact of a remote, long-term EEG tool (UnEEG 24/7 SubQ) for PwID and epilepsy.

**Method:**

We conducted six, 2-hour long workshops; three with people with mild intellectual disability and three with families/carers of people with moderate–profound intellectual disability. Brief presentations, easy read information and model demonstrations were used to explain the problem and device. A semi-structured guide developed by a communication specialist and art-based techniques facilitated discussion with PwID. For family/carers, active listening was employed. All conversations were recorded and transcribed. Artificial intelligence-based coding and thematic analysis (ATLAS.ti and ChatGPT) were synthesised with manual theming to generate insights.

**Results:**

Co-production included four PwID, five family members and seven care professionals. Three main themes were identified: (1) perceived benefits for improving seizure understanding, informing care and reducing family and carer responsibility to accurately identify seizures; (2) the device was feasible for some PwID but not all; and (3) appropriate person-centred communication is essential for all stakeholders to reduce concerns.

**Conclusions:**

The workshops identified key benefits and implementing barriers to SubQ in PwID.

The prevalence of epilepsy in people with intellectual disability (PwID; ~22%) is substantially higher than its prevalence in the general population (~0.5–1%).^1–3^ As well as having increased prevalence, PwID have statistically worse epilepsy-related outcomes than the general population: they have higher rates of treatment-resistant epilepsy, polypharmacy, comorbidity, misdiagnosis, preventable emergency department admissions, sudden unexpected death in epilepsy (SUDEP) and premature mortality.^3–6^ A key challenge contributing to these issues is the difficulty for PwID to convey their experiences to family members, carers and clinicians; day-to-day seizure detection in this population primarily relies on subjective assessment by these stakeholders, and behavioural, psychological and physiological events can be easily misinterpreted.^7^ The prescription of antipsychotic drugs off-label to manage challenging behaviours in PwID is common;^8,9^ the problem is that this can reduce seizure threshold and increase the risk of seizure activity in patients.^10^ Despite significant co-occurrence between epilepsy and intellectual disability, PwID and epilepsy are understudied and under-supported in epilepsy research and care,^11^ which can negatively affect their care. Given the prevalence of epilepsy in PwID, it is necessary to develop strategies and technologies to address these barriers systematically to ensure that PwID receive equitable access to healthcare.

## Challenges with seizure monitoring in PwID

Various factors contribute to the challenges identified with managing epilepsy in PwID. PwID experience unique barriers to undergoing standard epilepsy assessments. This includes a range of diverse challenges, from difficulties with PwID communicating about their own experiences, to being excluded from and participating in standard research trials. A particular issue is seizure detection. Electroencephalography (EEG) is a key tool for diagnosing and treating epilepsy by enabling the detection and analysis of seizure activity in the brain. A limitation with short-term EEG monitoring is that the likelihood of catching a seizure during a recording session is often low. Prolonged EEG monitoring can improve this likelihood, but often involves a prolonged stay in hospital, which can be especially difficult for PwID, who may be overstimulated by the unfamiliar environment and experience difficulty communicating their feelings or comprehending the reason for their admission to hospital.^12,13^ Remote EEG monitoring has the potential to reduce these barriers by enabling objective seizure data collection at home, particularly if the system is unobtrusive and does not interfere with daily activities.

## Exclusion of PwID in research

There are a variety of remote EEG monitoring systems being developed and evaluated, but few have considered their application for this population of patients.^14^ We conducted a review of remote EEG monitoring systems that identified only three studies that referred to PwID and only one that focused on remote EEG monitoring specifically for that population.^12,15^ This review highlighted the current lack of evidence for the use of remote EEG monitoring for seizure detection in PwID and the need for their inclusion in research to address the concerning data about their epilepsy-related health outcomes.^14^

As a vulnerable population, PwID are often excluded from clinical studies.^11,17–20^ A review of 180 randomly selected National Institute for Health and Care Research (NIHR) studies conducted in 2019–2020 found that 78% excluded PwID.^18^ This exclusion contributes to the significant health inequalities that PwID face;^21^ to ensure that PwID receive equitable care, their involvement in research and the generation of knowledge is essential.^19,20^ Challenges can include a lack of resources and training or ethical concerns about the potential to cause harm, but the exclusion of PwID from research or reliance on proxy respondents raises ethical issues relating to the silencing of the people who are most affected.^19,20^ Studies have found that PwID are interested in participating in research and identified the importance of trust in decisions to participate.^20^ To tackle challenges around health inequity and improve access to care for PwID, there is a need for more research that includes them as active co-designers and co-researchers.

## Rationale and research questions

New technologies that enable long-term, home-based EEG monitoring have the potential to enable objective seizure data to be captured for PwID. To determine how such technologies can be successfully implemented within this population, it is essential that PwID and their families and carers have a voice in and are included as co-designers in designing research. The purpose of such co-production workshops is to address this gap in access of PwID to necessary epilepsy care that respects their specific needs. This co-production work was undertaken to inform future evaluation of the UNEEG SubQ device in this population. The co-production focused on two key questions: (1) What do PwID and their families and caregivers perceive as potential benefits or barriers for adopting and using the UNEEG SubQ device for seizure monitoring in this population? and (2) How should future studies be designed to evaluate the device in this population to ensure that PwID and their families and caregivers are empowered and able to make informed participation decisions?

## Method

### Co-production design

**Fig. 1:**
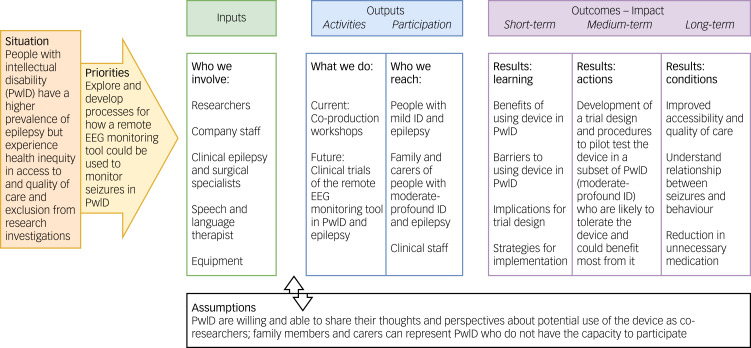
Wisconsin logic model. EEG, electroencephalography; ID, intellectual disability.

Co-production was used to centre PwID and their family members, carers and healthcare professionals as co-researchers. We chose to use such an approach to ensure that the voices of the people who would be using the device (PwID and epilepsy, as well as other key stakeholders responsible for their care) were integral in the determination of who the device would be appropriate for and how it could be implemented and improved to meet their needs.^22^ This approach has particular advantages when aiming to apply findings to improve decision-making around how to conduct research and implementation, to increase the involvement of vulnerable or excluded groups and to build trust with end users,^23–25^ making it well suited for our aims (Fig. 1).

### Ethics and consent statement

Research ethics approval for the co-production was obtained from the Newcastle University Faculty of Medical Sciences Research Ethics Committee (2926/51 222). Written consent was collected from all co-researchers before commencing workshops. This consent was provided in easy read format and confirmed co-researchers’ understanding of the workshops and their consent to participate and be recorded. Review of the patient and public involvement (PPI) information sheets and consent forms by PwID was supported by a speech and language therapist.

### Co-researchers and recruitment

PwID and family members, carers and healthcare professionals of PwID were recruited by two authors (S.M., R.S.) from a National Health Service (NHS) Trust. Clinical records of PwID with diagnosed epilepsy open to the authors’ clinical team were reviewed to identify eligible co-designers. An invite was sent to all eligible patients with mild intellectual disability and family members and residential care managers of those with moderate to profound intellectual disability to invite them to the workshops. A variety of stakeholders were invited to ensure that all relevant perspectives were included. The justification and rationale for dividing PwID into ‘mild’ and ‘moderate to profound’ intellectual disability is provided in Supplementary Appendix 1 available at https://doi.org/10.1192/bjo.2024.825.

### Intervention

The UNEEG SubQ device is a subcutaneous electrode that records EEG on two channels. It is minimally-invasive and can be implanted under local or general anaesthetic in a short out-patient appointment. A small disc sticks behind the patients’ ear above the implant and is attached via wire to a small, wearable recording device that captures the EEG data. The recording device can be removed if needed (e.g. if the patient is having a shower) and reattached easily. The device has been CE-marked (2019) and approved for an implantation period of up to 15 months, which enables high signal quality and data analysis to be collected over a long period of time. Data can be transferred from the recording device to clinicians by plugging it into a provided tablet.

Previous pilot and case study evaluations of the SubQ device have been conducted in the general population. These have demonstrated the feasibility and acceptability of device implantation and data capture, quality and completeness (averaging over 20 h per day) as well as its effectiveness at seizure detection compared to self-reported seizure diaries.^26,27^ One participant in these evaluations had mild intellectual disability and, with support from carers, tolerated the process easily, but to date, the device has not been purposefully trialled in a population of PwID.

### Procedure

Six 2 h workshops were conducted across three days in different locations in Cornwall, UK (pop: 538 000), with PwID in the mornings and family members and care professionals in the afternoons. Team members (including representatives from UNEEG, a surgeon and clinical researchers specialising in epilepsy and intellectual disability) delivered brief presentations and demonstrations. An easy read story (featuring a fictional patient, ‘Dan’), developed in collaboration with a speech and language therapist, was used to communicate complex details of the technology and surgical process with PwID. The speech and language therapist facilitated communication with the PwID and guided discussion using a semi-structured topic guide (Supplementary Appendix 2). PwID were asked about their own opinions and prompted with questions about how ‘Dan’ would feel about relevant aspects of the project to try and get a more generalisable perspective from the PwID, as well as their own. Arts-based methods, such as paper tablecloths that attendees could draw and write on, stoplight cards to pause discussions if PwID needed something to be better explained and drawings of response options, were used to facilitate the discussions, and feedback and sessions were audio recorded to ensure that no perspectives or comments were missed. A separate topic guide was developed to provide an initial structure for the focus groups with family members and care professionals and to ensure that over the course of each workshop, all of our questions were discussed (Supplementary Appendix 3).

### Analysis

#### Overview

Qualitative data from the focus groups was analysed using a theoretical thematic analysis approach in ATLAS.ti 23.4.0 for MacOS (ATLAS.ti Scientific Software Development GmbH, Berlin, Germany; https://atlasti.com),^28^ following Braun and Clarke's^29^ six phases of thematic analysis. The thematic analysis combined manual and artificial intelligence techniques to generate themes (Table 1). Before inputting any data into data analysis tools, the transcripts were anonymised by replacing all names with an identifier indicating the co-researcher type (e.g. PAR-1-PWID or PAR-2-CARER).

**Table 1:** Execution of Braun and Clarke's six phases of thematic analysis in the analysis of the co-production workshops^29^

Phase	Execution in this co-production
1. Familiarising yourself with your data	One author reviewed the focus group transcripts multiple times over a period of 6 months, including generating edited versions of the transcripts for artificial intelligence coding in ATLAS.ti that included only co-researchers’ thoughts and responses and excluded the focus group facilitators’ contributions.
2. Generating initial codes	Codes were generated using the ATLAS.ti ‘intentional artificial intelligence coding’ feature on the revised transcripts in two rounds.
3. Searching for themes	Stage 1: One author reviewed all of the original transcripts manually and theoretically generated themes based on two key questions. This was conducted separately for the workshops with people with mild intellectual disability and those with family and carers of people with moderate to profound intellectual disability before reviewing the artificial intelligence-generated codes and was not influenced by the results of the artificial intelligence analysis in Phase 2. Stage 2: ATLAS.ti artificial intelligence-generated codes were automatically grouped by ATLAS.ti. For each code grouping, all codes within that group were input to ChatGPT 3.5 with the prompt ‘Create a thematic summary of the following codes:’.
4. Reviewing themes	The themes generated by ChatGPT in Phase 3, Stage 2, were examined and refined manually. They were then compared with the manually generated themes from Phase 3, Stage 1.
5. Defining and naming themes	Based on the comparison of the manually and artificial intelligence-generated themes, the final set of themes was refined in relation to the questions.
6. Producing the report	The final analysis is included in the results and discussion of this paper.

#### ATLAS.ti coding

Coding of the data was conducted using the ATLAS.ti ‘Intentional artificial intelligence coding’ feature, which codes the transcripts based on user-provided research questions or aims. This feature works by using generative pre-trained transformer (GPT) large language models, trained on a large body of texts and with human input. The transcripts are divided into chunks by ATLAS.ti, which are then repeatedly passed through GPT models, accounting for a ‘window of attention’ of at least 100 characters (text surrounding the text being assessed, to provide context). ATLAS.ti then automatically groups the artificial intelligence-generated codes into pre-specified categories based on the research purpose or questions input by the researcher (Table 2). Details are provided in the ATLAS.ti 24 user manual.^30^ As the artificial intelligence coding feature codes all text without focusing solely on co-designers’ responses, the original transcripts were revised to remove all facilitator comments. To meet the communication needs of the PwID, many of the questions in those workshops were phrased in simple yes/no and multiple choice formats. This meant that the facilitator question had to be incorporated into the PwID's response to ensure sufficient context for the artificial intelligence coding. Text that was added in this way was clearly marked as not being the co-designers’ own contribution by bolding the text and putting it in square brackets.

**Table 2:** Research questions specified for the intentional artificial intelligence coding process

Question	Code category
What are the potential benefits for people with intellectual disabilities of an implantable device for seizure monitoring for people with epilepsy and intellectual disability?	Perceived benefits for PwID
What are the potential benefits for family members and carers of an implantable device for seizure monitoring for people with epilepsy and intellectual disability?	Perceived benefits for family/carers
What are the potential benefits for clinicians of an implantable device for seizure monitoring for people with epilepsy and intellectual disability?	Perceived benefits for clinicians
What are the potential barriers or issues to the use of this device for people with intellectual disabilities?	Potential barriers for PwID
What are the potential barriers or issues to the use of this device for family, carers and clinicians of people with intellectual disabilities?	Potential barriers for family, carers and clinicians
How do people with intellectual disabilities feel about the surgery to implant the device?	PwID's perceptions of surgery
What is the best way to communicate with people with intellectual disabilities about the device?	Communication with PwID
What types of patients with intellectual disability would this device be best suited for?	Target population
What do patients with intellectual disabilities think and feel about having and using the device?	PwID's perceptions of device

PwID, people with intellectual disability.

#### ChatGPT theming

ChatGPT 3.5 for MacOS (OpenAI, San Francisco, California, USA; https://chat.openai.com) was used to theme the ATLAS.ti analysis to gain another perspective on these codes using the prompt ‘Create a thematic summary of the following codes’. As with ATLAS.ti, ChatGPT uses a language model (GPT-3.5) to generate text. It has also been trained on a large data-set of texts and refined using human feedback.^31^ This process was conducted in two different ways: the prompt was applied to all of the codes as a whole and separately to all of the codes within each code category in Table 2. The ChatGPT themes and summaries were then compared (Supplementary Appendix 4).

#### Trustworthiness

To improve the credibility of the results, the author conducting the thematic analysis engaged extensively with the data over a long period of time, including participating in all focus groups and multiple reviews of the transcripts to prepare them for artificial intelligence analysis. In addition, the author manually developed preliminary theoretical themes relating to the two research questions based on an in-depth review of the transcripts before viewing the artificial intelligence-generated codes (Supplementary Appendix 5). The manually and artificial intelligence-generated themes were then compared to generate the final set of themes.

## Results

### Co-researchers

To ensure that we gathered a range of perspectives, a diverse group of 16 stakeholders participated in the various workshops. The co-researchers included four people with mild to moderate intellectual disability, five family members of people with moderate to profound intellectual disability (three parents, a brother and a sister-in-law) and seven care professionals of people with moderate to profound intellectual disability (four carers, a care home manager, an epilepsy nurse and an epileptologist).

### ATLAS.ti artificial intelligence codes

The ATLAS.ti intentional artificial intelligence coding process generated 842 codes. Of these, 82% of the codes (688/842) were only coded once and 97% (815/842) were coded five times or fewer. Ten codes were applied to ten or more quotations (Table 3).

**Table 3:** Codes applied to ten or more quotes

Code	Category	Number of quotes
Peace of mind	Perceived benefits for family/carers	47
Accurate data	Perceived benefits for clinicians	22
Reduced stress	Perceived benefits for family/carers	19
Increased safety	Perceived benefits for PwID	16
Improved monitoring	Perceived benefits for clinicians	14
Improved seizure management	Perceived benefits for PwID	14
Seizure monitoring	Perceived benefits for PwID	13
Discomfort	Potential barriers for PwID	12
Treatment planning	Perceived benefits for clinicians	11
Timely assistance	Perceived benefits for family/carers	10

PwID, people with intellectual disability.

### Themes

**Fig. 2:**
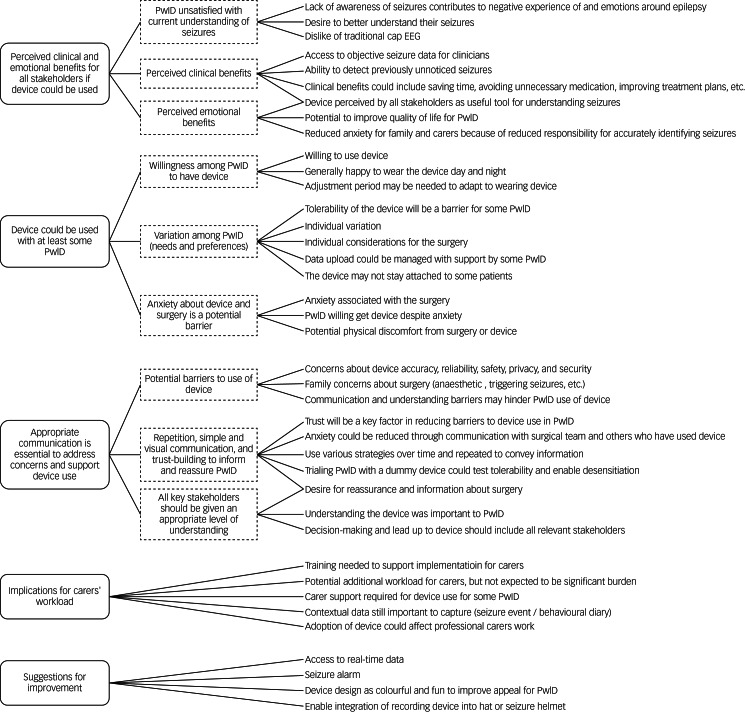
Thematic framework. PwID, people with intellectual disability; EEG, electroencephalography.

The thematic framework (Fig. 2) was developed by synthesising the independent artificial intelligence and manual analyses of the transcripts. Three main themes were identified: (1) all stakeholders perceived clinical and emotional benefits of the device; (2) the device was feasible for at least some PwID; and (3) appropriate communication is essential to addressing stakeholder concerns and supporting device use. There were also a couple of smaller themes around the potential implications for carers’ workloads and suggestions for how the device could be improved for this population.

#### Theme 1: perceived clinical and emotional benefits

Overall, all of the co-researchers thought that the SubQ device was a good idea and would be beneficial to patients, family, carers and clinicians. PwID reported that they were not always aware of when they have seizures, which some found distressing. One person with intellectual disability highlighted that they ‘would love nothing more than to understand what actually goes on’ with their epilepsy; another person with intellectual disability also felt that it would ‘help me a lot more to understand more about epilepsy and my medication and why I take it’. Several PwID and carers highlighted a dislike of traditional EEG cap recordings; the wires and the gel are unpleasant for many PwID. Families and care professionals agreed that having objective data would be ‘revelational’ and a ‘life changer’ for ‘two reasons: one, it will help give a better picture of what's going on for someone … and [two,] it takes a lot of responsibility off other humans to record what's going on’. Regular seizure identification and reporting is subjective, especially because many of PwID cannot communicate verbally about their experiences; one carer felt that there were ‘all sorts of things… being missed’. The potential of the device to capture previously unnoticed seizures, inform clinical care and avoid unnecessary medication was perceived as having the potential to improve quality of life not just of the patient but of the family members and carers as well.

#### Theme 2: device suitable for some PwID

All four of the PwID who participated in the workshops were willing (and in some cases eager) to have the device. For PwID more generally, key barriers to use that were identified were anxiety and tolerability. PwID co-researchers were ‘a bit worried’ and ‘anxious’ about the surgery but stated that they ‘would be fine if it happened’. They suggested several strategies to help manage this anxiety, which are captured in Theme 3. Most of the PwID's anxiety centred around the surgery; PwID were generally happy to wear the external device, with one person with intellectual disability stating that they ‘would wear it forever’, although another felt that although they ‘[did] like it, it will take time to get used to [because] it feels weird at first’. Wearing the device at night was also a potential issue raised by one person with intellectual disability, as it would depend whether the recording device was on the side of the head that they slept on. Although being personally fine with the device, the person with intellectual disability recognised that the device may not be for everyone: ‘some individuals are probably different compared to me so it all depends on the person’ and ‘not everybody wants to know how many seizures they have’. In addition to individual variation in PwID's perceptions and toleration of the device, there was variation in how much support they would require to use it; some patients with mild intellectual disability would be able to manage use of the device themselves with minor support, but many other PwID would require a carer to manage the device for them.

Family members and carers of people with moderate to profound intellectual disability thought that while many patients would be able to tolerate the device, some would not. One carer stated that ‘I've got 12 people now that it would work perfectly on. 12 people who would tolerate it. 12 people it would be helpful [for]. All of these are absolutely a huge SUDEP risk. There's only one that I would think would probably scratch it out from the inside of his head’. The wire on the external recorder was a particular issue for tolerability. Family members of one person with intellectual disability felt that although the ‘principle of it is absolutely fantastic … the device as it stands will still have a restricted application in clients like [name] … because there's just no way somebody like him would tolerate this wire’. Even for patients who may not deliberately try to remove the device or pull the wire, a carer highlighted that it would be frequently disconnected ‘because we have some quite wiggly individuals’. Another factor that could influence tolerability was how the device looked, with another carer providing the example of a person with intellectual disability who is ‘very particular about how he looks [and who] would certainly worry [because] it is not something that he particularly likes people to know about’.

#### Theme 3: appropriate communication is essential to address concerns and support use

Communication with all stakeholders at an appropriate level is key to mitigating barriers to use, particularly around anxiety and tolerability. PwID co-researchers wanted ‘some information about it before’ about ‘good and bad things’. Demonstrations, easy read picture stories and videos of patients with the device talking about their experience could help their understanding of the device and provide reassurance. There were mixed feelings about cartoons and animation in videos, with some co-researchers highlighting this as a good option to engage PwID but one carer reporting that ‘a lot of ours have responded better to actual people than [animated] videos’. There was also an emphasis on the importance of repetition, both to improve the comprehension of PwID and to improve tolerability of the device through desensitisation.

Building trust was important for both patients and families. To help reduce surgery anxiety for PwID, co-researchers suggested seeing the hospital environment in advance, having a support person with them (family, friend or carer), and ‘talking to the surgeon … so they can explain it a bit more and calm the person down … will probably make [the PwID] realise okay they are not bad people and then they will realise that they can trust [the surgeon]’. The family member co-researchers who attended the workshops had many questions about the safety, accuracy and previous experiences of patients using the device. Detailed information about benefits and risks could help build trust with families and enable best-interest decision-making. Co-researchers also highlighted the importance of having all relevant stakeholders understanding and supporting the process.

#### Other themes

Other themes that were brought up by co-researchers included the implications of the device for carers’ work. The carer co-researchers generally felt that ‘[it would] probably [be] around the same level [of work]’ – one specified that they thought ‘it will save so much time in the long run – that five minutes for [uploading data] is fine’ – and that ‘there wouldn't be much training’ needed to operate the device. However, a care home manager raised the concern that ‘staff could get complacent that it's all going to be recorded [by the device] and not realise … [that] there is still an amount of time you need to action things when seizures are going on’, so some training to ensure carers understand the role of the device in relation to standard care would be important.

There were also several suggestions from family and carer co-researchers about how the device itself could be improved for this population. For example, changing the design in terms of functionality – ‘do you think with time it would be able to be wireless?’ – and aesthetic – ‘something that is brightly coloured or something that can go into a brightly coloured case is more attractive than something that looks clinical’. Another suggestion was that if the device could be incorporated into or attached to hats or epilepsy helmets, it might be an easier way to ensure that more PwID could tolerate the devices for sufficient periods of data collection.

## Discussion

### Principal findings

This co-production outlines the co-research process used to ensure that PwID with epilepsy and other key stakeholders had a voice in generating the key outputs of this work – that is, identifying benefits, barriers and strategies for how to use and evaluate the SubQ device in this population. Overall, PwID, family members and care professionals felt that the device would be a valuable tool and identified key clinical and emotional benefits, such as improved detection and understanding of seizures and reduced responsibility on observers. The PwID co-researchers were all willing to have the device and felt that they would be able to wear it for long periods of time. The main barriers to the use of the SubQ device in PwID were tolerability and anxiety. As it is currently designed (particularly because of the wire), the device would not be tolerable for all PwID. Both PwID and family members expressed concerns and anxiety about the device; PwID were primarily anxious about the surgery, while family members desired detailed information of the device's safety and potential side-effects. In terms of future trial design and clinical implementation, co-researchers identified several strategies for addressing these barriers. Desensitisation and trialling the tolerability of the external recorder before surgical implantation would help to determine whether the device is suitable for a particular person with intellectual disability. Anxiety can be mitigated by ensuring that all stakeholders feel sufficiently well informed about the device – for PwID, this should include repetition of the information over time via various formats (e.g. easy read stories, videos of others’ experiences with the device, demonstrations, modelling) – and by establishing trust and comfort with the clinical (and particularly the surgical) team and environment.

### Strengths and limitations and benefits and challenges of co-production

A strength of the co-production was our effort to centre people with lived experience as co-designers through the workshops. Engaging these stakeholders not just as participants but as active contributors to the development of strategies and processes for future research and implementation of the device in this population will help to improve its accessibility and acceptability. We included a diverse group of stakeholders (including PwID, their family and carers and healthcare professionals) that helped capture the different experiences and perspectives of everyone involved. This also helped us to determine which PwID personality makeup this device might be most suitable for.

As intellectual disability can present challenges for communication, we made a particular effort to ensure that PwID felt empowered and understood in the workshops. A speech and language therapist experienced with working with PwID facilitated the discussions using verbal and visual methods (drawings and demonstrations) and the PwID's family member or carer was brought in to assist with communication if there were any difficulties in our comprehension of the PwID's contributions. The workshops had a large number of stakeholders from the research team. This was felt necessary to ensure that the right people were in the room to address any specific questions relating to epilepsy, the device or the surgery; however, it was recognised that this had the potential to be overwhelming for PwID. To mitigate this, we had an initial period of conversation to introduce everyone and talk about topics the PwID were interested in before moving onto the topic of the workshops.

One of the limitations of the co-production was that there was a relatively small number of PwID co-designers. This likely limited the generalisability of perspectives captured. We attempted to mitigate this to some extent by using an easy read story and asking PwID co-researchers what they thought a fictional patient (‘Dan’) would feel about the device and surgery. Several PwID co-researchers did also highlight that while they wanted the device, they did not think that all PwID would and that it would depend on the individual. However, it would have been beneficial for the analysis to have perspectives from PwID who did not want the device as well, to identify what factors or characteristics may have contributed to this preference.

Another potential limitation of the co-production was that the thematic analysis was conducted using artificial intelligence tools, which risks misinterpreting data. This was mitigated by conducting an independent manual thematic analysis of the transcripts and comparing and synthesising the data; however, this was only conducted by one author, and although that author had prolonged involvement with the transcripts, a full manual coding process was not feasible owing to time and resource constraints of this co-production activity. This limitation was mitigated through the use of artificial intelligence as an independent means of coding and theming the data. As artificial intelligence becomes more sophisticated, its applications for thematic analysis are being increasingly explored; while there are risks of misinterpretation or an overly strong emphasis on content rather than theme, when used in conjunction with researchers, it has potential benefits for facilitating coding and suggesting themes that may otherwise have been missed.^32–34^ The use of artificial intelligence in the context of this co-production analysis was a contribution to this early field of literature aiming to understand the benefits and challenges of its application to thematic analyses.

### Implications for future research

This project focused on co-production work because of the importance of PPI to ensure that future research is conducted in a way that is ethical and centres the needs and lived experience of PwID. Co-production approaches are a burgeoning area of social scientific and health research,^35–40^ although projects often fail to locate PwID as research leaders.^41^ Despite the growing emphasis on PPI and co-production in research, there is still a lack of defined approaches and processes for conducting such collaboration; such an approach would inevitably not meet the particular needs and contexts of different research aims.^25,42^ Broadly, a co-production model of research emphasises the collaborative effort of researchers and people with lived experience to generate knowledge based on principles of power sharing and recognition that people with lived experience have unique information and skills to shape the design of research.^23,42^

The workshops provided us with valuable insights into the perspectives of key stakeholders around the device, the potential barriers to its use and strategies for how those barriers could be mitigated. This will enable future trials to be conducted in a way that is ethical and centres the needs and lived experience of PwID and to avoid potential issues with conducting a large-scale co-production and future clinical adoption. Although such focused co-production work is not always feasible, we would recommend it where possible, and particularly for patient populations with unique needs and abilities that are often excluded from research or those that experience other health inequities.

Based on the findings generated from the workshops, we are planning a pilot study to evaluate the use, acceptability and tolerability of the SubQ device in patients with moderate to profound intellectual disability. Family members and carers highlighted that the device could be particularly beneficial for people with moderate to profound intellectual disability who do not have the capacity to communicate their experiences and for whom seizures could be confused with challenging behaviour. We designed the planned study to focus on this population, in line with one of the co-researchers’ statement that ‘If you road test with learning disabilities first you're going to get it right for the general population’. Co-researchers also shaped the information, consenting and surgical procedures that will be followed. As all stakeholders emphasised that the best way of doing things will vary depending on the individual, the co-production will be set up to enable flexibility and help mitigate risk – for example, provision of information in a variety of forms, the option for local or general anaesthetic and the opportunity to assess tolerability before committing to the surgery.

More generally, this co-production provided an example of how artificial intelligence coding tools can be used in thematic analysis. We found that, particularly for focus groups, a lot of preparatory work needed to be conducted on the transcripts. The artificial intelligence coding technique did not discriminate between facilitators and co-designers when generating codes, but as we did not want to analyse what we had said when describing the intervention or asking questions, the transcripts had to be modified to include only co-researcher contributions. The artificial intelligence coding technique seemed to prefer generating new codes for most quotations rather than generating codes that could be applied to similar sentiments throughout the transcript. This resulted in a large number of codes, most of which were only applied to one quote. ChatGPT facilitated the process by grouping and summarising these codes thematically, but the comparison of the artificial intelligence-generated themes and the manually generated themes determined that there were some areas where the artificial intelligence had misunderstood the text or generated a result that was irrelevant to the aim of the research. Combining artificial intelligence and manual techniques improved the speed of the analysis and helped mitigate the limitations of each method; the artificial intelligence analysis was thorough and did not bring pre-conceptions to the coding, while the manual analysis ensured that the final thematic framework focused on the research-relevant themes.

PwID are often excluded from research because of perceived difficulties with capacity and consent processes or a lack of training and resources.^18^ This co-production process helped bridge that gap by including PwID and other key stakeholders as experts by experience to determine how best to design inclusive studies that meet their unique needs and improve equitable access to care.^18^ This co-production explored the insights generated by PwID, family members and care professionals as co-researchers around using and evaluating a subcutaneous long-term EEG monitoring device in this population. Conducting these co-production workshops informed the design of a pilot study to evaluate the safety, acceptability and impact of the SubQ device in PwID. This process was extremely beneficial and we would recommend a similar process for other studies wanting to include PwID in clinical research. If this is not feasible, co-researchers highlighted particular strategies for informing patients and obtaining consent or assent, including the repeated use of various visual strategies for communication (such as demonstrations, videos and easy read stories with pictures) and building trust by providing opportunities for PwID to meet with the relevant clinicians.

## Supporting information

Meinert et al. supplementary material Meinert et al. supplementary material

## Data Availability

The data-sets generated and analysed during the current study are not available owing to them containing information that could compromise co-researcher privacy/consent.
