# Supplementary material for: Subcutaneous electroencephalography monitoring for people with epilepsy and intellectual disability: co-production workshops
Source: BJPsych Open. 2024 Dec 13;11(1):e3. doi: 10.1192/bjo.2024.825 (PMC11733491; doi:10.1192/bjo.2024.825)
Supplement: Meinert et al. supplementary material [file S2056472424008251sup001.docx]

# Appendices

## Appendix 1. Rationale for dividing patients by mild and moderate-profound ID

1. People with moderate to profound ID are defined by qualitatively significantly higher levels of impairments. Where people with mild ID have near independent lives with some or minimal support, those with moderate to profound ID tend to be supported and supervised at all times.
2. Impairments such as communication difficulties, and thus making informed choices or engaging in meaningful participation, is significantly challenging for people with moderate to profound ID. They require lifelong advocacy on health and social choices. People with mild ID can make informed choices on most day-to-day matters and can be supported to provide a personal view on management and treatment choice if supported with suitable tools to simplify communication.
3. Epilepsy, possibly due to disturbed brain function, is present in 30-50% of the moderate to profound ID group as compared to 8-12% in the mild ID population and 0.5-1% in the general population.

## Appendix 2. Sample topic guide for PwID

1. *(How do you think Dan feels about having an implant?)*
2. How would you feel about perhaps having an implant?
3. Do you want an implant?
4. *(Do you think Dan would worry about having an implant? Why?)*
5. Is there anything that worries you about having an implant?
6. Do you use a phone/fitbit/smartwatch?
7. What do you think about the implant giving doctors more information about your seizures? Do you think it would be useful for the doctors to know more about your seizures?
8. Do you want to know more about your seizures?
9. How do you think the implant might help you? Do you think it could help you understand your seizures better?
10. *(How do you think Dan feels about wearing the recorder?)*
11. How would you feel about wearing the recorder?
12. *(How long do you think Dan could wear the recorder for?)*
13. How long do you think you could wear the recorder for?
14. *(How do you think Dan feels about wearing the recorder at night?)*
15. How would you feel about wearing the recorder at night? When you’re asleep?
16. What is the best way of telling other people about this implant idea?
17. Is a video a good idea? What about a demonstration? What about easyread information?

##

## Appendix 3. Sample topic guide for families, carers, and HCPs

There are a few different areas we would like to discuss. We are interested in your perspective on the potential benefits and challenges of using this technology with people with moderate to profound learning disabilities - both in terms of standard clinical care and research. We are also curious about how the use of this technology with people with moderate to profound learning disabilities would affect you as family members, caregivers, and care professionals.

*Perspective on behalf of people with moderate/profound learning disabilities*

1. What initial thoughts do you have about the use of this technology with people with moderate to profound intellectual disability?
2. How would you explain this device to someone with moderate to profound learning disabilities if they were offered it to monitor their epilepsy?
3. What concerns, if any, do you have about getting informed consent / assent from patients?
4. How do you think people with moderate to profound learning disabilities would feel about having the surgery?
5. The surgery can be conducted under local (with or without sedation) or general anesthetic - what are your thoughts about which would be better for people with moderate to profound learning disabilities?
6. How would you suggest explaining the day to day use of the device to someone with moderate to profound learning disabilities?
7. How could we facilitate this process?
8. How well do you think people with moderate to profound learning disabilities would tolerate wearing the recording device?
9. How could any potential issues with this be improved / avoided?
10. What other issues do you anticipate regarding use of the device?
11. How much time would you foresee someone with moderate to profound learning disabilities using the device for each day?
12. Would daytime or nighttime be most difficult for people with moderate to profound learning disabilities to wear the device?

*Perspective on behalf of family members / caregivers*

1. What, if any, challenges or issues do you expect the use of the device to have for caregivers?
2. What, if any, benefits do you expect the use of the device to have for caregivers?
3. How closely are people with moderate to profound learning disabilities typically supervised? What level of support can we expect from caregivers?
4. What strategies would you use if you were trying to support someone with moderate to profound learning disabilities who was reluctant to use the device?
5. How often could you interact with the device - ie. to record seizures as they happen (seizure diary by pressing button on wearable)?

*Perspective on behalf of patients’ health care team*

1. What concerns would you have about suggesting this device for someone with moderate to profound learning disabilities?
2. What benefits do you foresee from the use of this device for people with moderate to profound learning disabilities and epilepsy?

##

## Appendix 4. Two rounds of ChatGPT theming based on the codes

| **ATLAS.TI theme** | **ChatGPT theme based on codes**  **(theme codes included)** | **ChatGPT theme based on codes**  **(all codes included)** |
| --- | --- | --- |
| Perceived Benefits for clinicians | Accurate Data and Diagnosis | Access to accurate seizure data |
|  |  | Better understanding of seizure patterns |
|  | Improved Treatment and Care | Improved accuracy in diagnosis and treatment planning |
|  |  | Timely intervention and treatment adjustments |
|  | Enhanced Communication and Collaboration | Enhanced collaboration |
|  | Efficiency and Streamlining |  |
|  | Objective and Real-Time Monitoring |  |
|  | Understanding and Education |  |
|  | Research Potential and Data Value |  |
| Perceived Benefits for family/carers | Access to Information and Monitoring | Improved understanding of epilepsy and seizures |
|  | Improved Care and Support | Better care and support |
|  | Reduced Burden and Anxiety | Reduced anxiety and stress |
|  |  | Increased peace of mind and reassurance |
|  | Enhanced Understanding and Awareness |  |
|  | Improved Communication and Response | Timely assistance and support during seizures |
|  | Enhanced Safety and Security |  |
|  | Personalization and Tailored Support |  |
| Perceived Benefits for PwID | Accurate Monitoring and Management |  |
|  | Improved Quality of Life | Improved quality of life |
|  | Enhanced Safety and Security | Enhanced seizure management and support |
|  | Support and Assistance |  |
|  | Empowerment and Independence | Increased independence and empowerment |
|  | Communication and Understanding | Improved communication with healthcare professionals |
|  | Technological Advancements |  |
|  |  | Potential for better treatment outcomes and medication management |
| Potential Barriers for family, carers, and clinicians | Access and Implementation Challenges | Additional workload for carers and clinicians |
|  | Accuracy and Reliability Concerns | Concerns about device reliability and accuracy |
|  | Communication and Coordination Challenges | Challenges in interpreting and responding to alerts |
|  | Privacy and Security Concerns | Privacy and data security concerns |
|  | Training and Education Needs | Need for training and education on device usage and interpretation |
|  | Technical and Operational Difficulties |  |
|  | Uncertainty and Worry |  |
|  | Cost Constraints |  |
|  | Complexity and Limited Understanding |  |
| Potential Barriers for PwID | Acceptance and Adjustment | Discomfort and anxiety related to surgery and device usage |
|  | Physical Discomfort and Intolerance |  |
|  | Communication and Understanding |  |
|  | Surgical Concerns and Invasiveness |  |
|  | Privacy and Invasion of Privacy | Concerns about invasion of privacy and discomfort during activities |
|  | Maintenance and Ongoing Support | Need for ongoing support and education |
|  | Tolerance and Adaptation |  |
|  | Uncertainty and Understanding | Fear and uncertainty about the effectiveness of the device |
|  | Activity Restrictions and Limited Entertainment |  |
|  | Representation and Advocacy |  |
| PwID perceptions of device | Appreciation and Positive Perceptions | Enthusiasm tempered by concerns about discomfort and invasion of privacy |
|  | Desire and Unwillingness | Mixed feelings, varying from appreciation to skepticism |
|  | Individual Variation | Individual variation in willingness to wear the device |
|  | Understanding and Questions |  |
|  | Capability and Functionality |  |
|  |  | Desire for better understanding and support |
|  |  | Openness to trying the device but with reservations about its impact on daily life |
| PwID perceptions of surgery | Acceptance and Openness | Willingness to try if it leads to better seizure management and understanding |
|  | Anxiety and Fear | Anxiety and fear, mixed with curiosity and interest |
|  | Mixed Feelings and Varying Preferences | Varying preferences and levels of comfort with the idea of undergoing surgery |
|  |  | Skepticism about the benefits versus the discomfort and potential risks |
|  | Desire for Information and Support | Desire for reassurance and information about the procedure |
|  | Comfort and Reassurance |  |
|  | Interest and Curiosity |  |
|  | Supportive Environment and Positive Perception |  |
|  | Practical Considerations and Comfort Levels |  |
| Target population | Autism and Learning Disability |  |
|  | Desire for Better Understanding |  |
|  | Interest in Seizure Monitoring |  |
|  | Need for Assistance in Seizure Management |  |
|  | Openness to Monitoring |  |
|  | Physical Intolerance and Intellectual Disability |  |
|  | Regardless of Intellectual Functioning |  |
|  | Regular Seizure Monitoring |  |

##

## Appendix 5. Summary of manually-generated themes

| **Theme** | **Description** |
| --- | --- |
| Lack of awareness of seizures contributes to negative experience of and emotions around epilepsy | PwID are not always aware of their seizures; this contributed to their experiences with epilepsy which were variably characterised as unwell, sad, angry, and scary but universally negative |
| Dislike of traditional cap EEG | PwID disliked the feeling of the cap EEG and the fact that it prevented them from doing anything while being monitored |
| Desire to better understand their seizures | PwID disliked the uncertainty of not knowing about their seizures and wanted to know about how often and when they were having seizures |
| Device perceived as useful tool for understanding epilepsy | PwID expected that the device would help them and their doctors understand their seizures better |
| Willing to use device | All PwID co-researchers would get the device if offered |
| Individual variation | Despite all being willing to get the device, PwID felt that not everyone would want it (e.g. people might not want to know more about their seizures) |
| Understanding the device was important to PwID | PwID wanted to understand the benefits and risks of the device |
| Anxiety associated with the surgery but not considered a barrier to having device | PwID mostly felt anxious at the prospect of the surgery but also stated that this would not prevent them from having it |
| Use various strategies over time and repeated to convey information | PwID liked the explanation with demos and materials and would like images of the healed operation and a video of someone with the device sharing their experience, but felt the information should not be presented all at once so as not to be overwhelming; family members and carers generally concurred that easy read materials with pictures, social stories, and particularly videos would be useful (difference of opinions on cartoons) |
| Strategies to reduce anxiety | Anxiety about the surgery could be reduced or avoided by explaining the implant and procedure; in addition to the general communication strategies included in the previous theme, building trust by meeting the surgical team and experiencing the surgical environment ahead of time could help reduce anxiety as could having someone accompany them. PwID wanted information about the benefits and risks but not too much graphic detail about the surgery. |
| Individual considerations for the surgery | Use of general or local anaesthetic will depend on the individual, dissolvable vs. normal stitches, etc. |
| Generally happy to wear the device day and night | PwID mostly expressed concerns about discomfort, except for one person who felt wearing the device while sleeping would depend what side it was on, but PwID did raise concerns about dropping the device |
| Data upload could be managed with support by some PwID | Some PwID were comfortable using technology, but might require a reminder to plug in the device for data upload and charging |
| Families desired a detailed understanding of how the device operates and safety and efficacy data | Family members and carers asked a variety of questions about the use, implanation, sensitivity, accuracy, and safety of the device; this information would need to be provided to legal decision-makers to provide reassurance of safety |
| Ability of device to detect previously unnoticed seizures and provided more detailed data would have significant benefits | Carer reports are subjective (based on interpretation of physiological and behavioural signs) and PwID cannot necessarily communicate their experience |
| Clinical benefits | These could include saving time, avoiding unnecessary medication, improving treatment plans, providing data about warning signs of mortality |
| Reduced anxiety for family and carers | Reduced anxiety was anticipated by family and carer co-researchers because of reduced responsibility for accurately identifying seizures |
| Adoption of device could affect professional carers work | Potential benefit by enabling training and learning about individual symptoms for seizure detection, potential risk that staff get complacent about recording data |
| Contextual data still important | A seizure / behavioural / trigger diary should still be kept to supplement the EEG data |
| Tolerability of the device will be a barrier for some PwID | People with profound ID would generally be unaware, unbothered, or have insufficient mobility to remove, but some patients would not tolerate the device (particularly the wire) |
| The device may not stay attached to some patients | Some patients move a lot, having to hunt down the device and reattach it could be an issue for carers |
| Trialling PwID with a dummy device could test tolerability and enable desensitisation | Some patients may be able to build up tolerance to device if desensitised to it slowly; if not, it provides a means of testing tolerability before conducting surgery |
| Trust will be a key factor in reducing barriers to device use in PwID | Modelling by a trusted person could assist with desensitisation, building trust with surgeons could reduce anxiety about surgery |
| Decision-making and lead up to device should include all relevant stakeholders | Ensuring that family members and carers are onboard will facilitate implementation and building understanding in PwID, include epilepsy specialist nurses as well |
| Future suggestions | Seizure alarm, live data availability, make device colorful and fun to make it appealing to PwID, enable integration of device into hat or helmet |
